# Uncover the identity of obstruction on the Achilles tendon

**DOI:** 10.1186/1757-1146-7-S1-A43

**Published:** 2014-04-08

**Authors:** Ryuta Kinugasa, John A Hodgson, V Reggie Edgerton, Shantanu Sinha

**Affiliations:** 1 Department of Human Sciences, Kanagawa University, Yokohama, Kanagawa, 2218686, Japan; 2 Department of Integrative Biology and Physiology, University of California Los Angeles, Los Angles, California, 90095, USA; 3 Department of Radiology, University of California San Diego, San Diego, California, 92121, USA

## Introduction

A mechanism must operate directly on the Achilles tendon which in effect introduces an obstruction to the outward movement of the Achilles tendon, but the features of this obstruction (what is the fundamental nature and cause and its physiological significance) are largely unexplored. We hypothesized that the obstruction arises from the differences in mechanical properties between muscle contractile tissue and non-contractile tissue. A possibility is that the pennate arrangement of muscle fibers results in a mechanical system which applies force vectors perpendicular to the muscle fiber axis, similar to that described for the action of intercostal muscle on the rib cage. The distal region of soleus (Sol) muscle has an unipennate arrangement, with fibers oriented between the posterior aponeurosis and anterior surface of the muscle. Although this configuration can constitute a constraint to the posterior movement of the Achilles tendon, the Kager’s fat pad, being non-contractile tissue, will be unable to actively develop any force and – render it mechanical incapable of constraining the movement of the tendon. If our hypothesis is true, the action of obstruction should be strongly synchronized to that of the tip of the Sol muscle as the ankle rotates.

## Methods

The anatomical location (x and y coordinates) and tissue movement (velocity) of Achilles tendon inflection point, which corresponds to the obstruction, and also those of the extremity of Sol distal edge were determined during passive and active contractions using MRI (n=6). A simple geometrical model was used to investigate how the position of the obstruction influences force and velocity gains.

## Results and discussions

**Figure 1:**
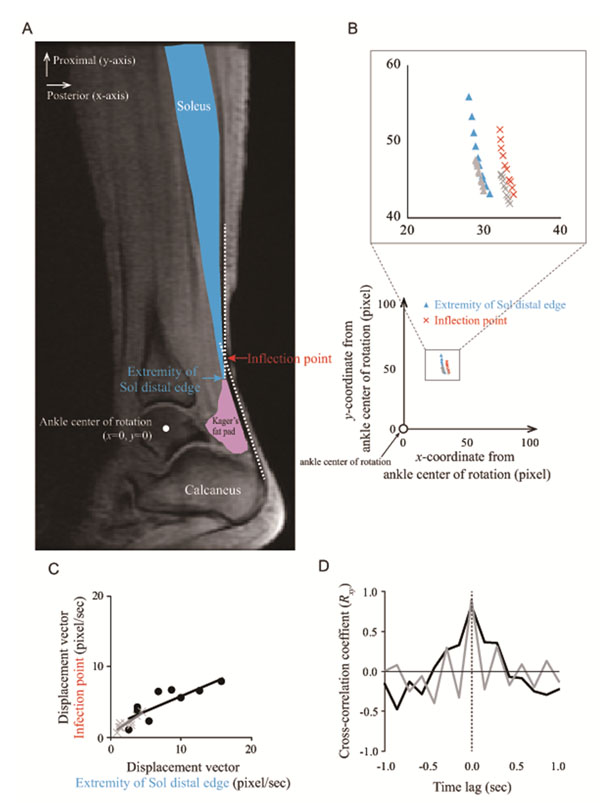
The synchronized movement between the inflection point and extreme distal edge of soleus (Sol) muscle during muscle contraction.

With increasing ankle angle, inflection point and extremity of the Sol distal edge moved in proximal and anterior directions (Figure 1B). The displacement vector, which implies a magnitude and direction, of the inflection point during ankle rotation was significantly correlated with the extremity of Sol distal edge (Figure 1C). A remarkable similarity of the cross-correlation coefficient was also found (Figure 1D). When the ankle initiated the plantarflexion force exertion, the Kager’s fat pad moved posteriorly, but the Sol muscle moved anteriorly. These results can be interpreted as direct evidence that the Sol muscle constrained the posterior movement of the Achilles tendon as ankle rotated, while the Kager’s fat pad did not. The gain would depend on the location of the obstruction relative to the ankle center of rotation. A more distal located obstruction resulted in variable gain over the range of motion; reduced force gain but increased velocity gain at high angles of plantarflexion.

## Conclusions

The Achilles tendon obstruction is likely to be emerged the location of boundary region between the Sol muscle and Kager’s fat pad when ankle positioned plantarflexion. Further, obstruction can provide a means of managing the tradeoff between force and velocity inherent in a finite power source and may effectively emerge in a location of terminal part of a joint such as foot or hand due to responsible for quicker movement rather than larger force exertions.

